# Effects of temperature and UVR on organic matter fluxes and the metabolic activity of *Acropora muricata*

**DOI:** 10.1242/bio.026757

**Published:** 2017-08-15

**Authors:** Lucile Courtial, Christine Ferrier-Pagès, Stéphan Jacquet, Riccardo Rodolfo-Metalpa, Stéphanie Reynaud, Cécile Rottier, Fanny Houlbrèque

**Affiliations:** 1 Sorbone Universités, UPMC, 4 Place Jussieu, 75252 Paris Cedex 05, France; 2 Centre Scientifique de Monaco, Equipe Ecophysiologie, 8 Quai Antoine 1er, 98000, Monaco (Principality); 3 UMR ENTROPIE (IRD, Université de La Réunion, CNRS), Laboratoire d'Excellence CORAIL, BP A5, 98848, Nouméa Cedex, New Caledonia; 4 INRA, UMR CARRTEL, 75 avenue de Corzent, 74200 Thonon-les-Bains, France

**Keywords:** *Acropora muricata*, Thermal stress, UV radiation, Climate change, Organic matter fluxes, Microbial loop

## Abstract

Coral bleaching events are predicted to occur more frequently in the coming decades with global warming. The susceptibility of corals to bleaching during thermal stress episodes depends on many factors, including the magnitude of thermal stress and irradiance. The interactions among these two factors, and in particular with ultra-violet radiation (UVR), the most harmful component of light, are more complex than assumed, and are not yet well understood. This paper explores the individual and combined effects of temperature and UVR on the metabolism of *Acropora muricata*, one of the most abundant coral species worldwide. Particulate and dissolved organic matter (POM/DOM) fluxes and organic matter (OM) degradation by the mucus-associated bacteria were also monitored in all conditions. The results show that UVR exposure exacerbated the temperature-induced bleaching, but did not affect OM fluxes, which were only altered by seawater warming. Temperature increase induced a shift from POM release and DOM uptake in healthy corals to POM uptake and DOM release in stressed ones. POM uptake was linked to a significant grazing of pico- and nanoplankton particles during the incubation, to fulfil the energetic requirements of *A. muricata* in the absence of autotrophy. Finally, OM degradation by mucus-associated bacterial activity was unaffected by UVR exposure, but significantly increased under high temperature. Altogether, our results demonstrate that seawater warming and UVR not only affect coral physiology, but also the way corals interact with the surrounding seawater, with potential consequences for coral reef biogeochemical cycles and food webs.

## Abstract (precis)

**Summary:** This study explores the individual and combined effects of temperature and UVR on the metabolism of *Acropora muricata*, and on the organic matter release and degradation by the associated bacteria.

## INTRODUCTION

Tropical marine ecosystems, including coral reefs, harbor more than 30% of the marine biodiversity (Doney et al., 2012), and provide goods and services to almost one billion people every year (Moberg and Folke, 1999; Wilkinson et al., 1999). However, they are currently threatened by climate change-induced increase in sea surface temperature (Nicholls et al., 2007), and in the incident flux of ultra-violet radiation (UVR, 280-400 nm) (Häder et al., 2007). This later increase is due to the effects of global warming on the stratospheric circulation and to a greater water stratification (Watanabe et al., 2011), leading to a deeper penetration of UVR in the water column (Vodacek et al., 1997).

The effects of rising sea surface temperature on coral physiology have already been well studied. Since most corals live at or near their threshold of temperature tolerance (Hoegh-Guldberg, 1999), thermal stress induces coral bleaching (i.e. loss of photosynthetic symbionts and/or chlorophyll content) and reduces coral photosynthesis and calcification (Hoegh-Guldberg, 1999). The coral response is, however, species specific, depending on the symbiont clade associated to the coral species (Wham et al., 2017), or the energetic reserves of the host tissue (i.e. lipid and protein content) (Fitt et al., 2009). It is also influenced by a myriad of environmental factors, including the level of UVR received by corals. Although UVR is highly mutagenic and enhances cell oxidative state, especially under elevated temperatures (Häder et al., 2007; Sharma et al., 2012), the combined effects of UVR and temperature on coral physiology remain poorly understood because of the complexity of the interactions between these two factors (Courtial et al., 2017; D'Croz and Maté, 2002; D'Croz et al., 2001; Ferrier-Pagès et al., 2007; Fitt and Warner, 1995; Lesser and Farrell, 2004; Lesser et al., 1990). Indeed, while no change was observed on the photosynthetic/autotrophic capacities of *Porites lobata* or *Turbinaria reniformis* under the combined stressors (Courtial et al., 2017; D'Croz et al., 2001), these capacities were strongly affected in *Montastrea annularis* and *Pocillopora damicornis* (Courtial et al., 2017; D'Croz and Maté, 2002; Fitt and Warner, 1995). The scarcity of experimental studies in this field does not allow good predictions of the combined effects of UVR and temperature on coral physiology. More studies are thus needed to better understand the species-specific response to these factors, and the mechanisms underlying coral susceptibility to thermal stress.

Two other underestimated aspects of thermal and UVR stress on coral biology concern the changes in organic matter (OM) fluxes (uptake and/or release of OM by corals) and recycling by the associated bacteria. Under healthy conditions, corals can release half of the photosynthetically fixed carbon and nitrogen into the surrounding reef waters in the form of mucus, i.e. dissolved and particulate carbon (DOC and POC, respectively) and nitrogen (DON and PON, respectively) (Crossland et al., 1980; Davies, 1984). OM is then degraded by prokaryotes through their extracellular enzyme activity (EEA), and is used for bacterial growth (Cunha et al., 2010), or it enters into the recycling pathways of carbon and nitrogen (Wild et al., 2004). OM therefore supports pelagic and benthic production, and plays a major role in the nutrient cycles and trophic structure of the whole reef ecosystem (Bythell and Wild, 2011). Elevated temperature, UVR and other stressors can, however, indirectly alter the quality and quantity of OM released by corals (Niggl et al., 2008; Tremblay et al., 2012; Wooldridge, 2009), and change the associated bacterial diversity (Ainsworth and Hoegh-Guldberg, 2009), likely affecting OM degradation rates. Although few studies have investigated OM fluxes in healthy and thermally stressed corals (Fonvielle et al., 2015; Grottoli et al., 2006; Levas et al., 2015; Niggl et al., 2008; Tremblay et al., 2012; Wooldridge, 2009), the effects of UVR on these fluxes remain unknown. As far as we know, the impact of elevated temperature and/or UVR on the enzymatic activities of mucus-associated bacteria has also never been investigated in tropical corals. The only knowledge on this subject comes from studies performed on water column bacteria from temperate and cold systems (reviewed in Cunha et al., 2010). They showed that bacterial enzymatic activities can be enhanced by temperature and repressed by UVR because of direct enzyme photolysis. Understanding how thermal and UVR stresses alter microbial degradation of coral OM and microbial growth will improve our understanding on future changes of the reef biogeochemical cycling, remineralization pathway and reef trophic structure.

The purpose of this study was to address some existing knowledge gaps regarding the effects of thermal stress, UVR and their combination on the quality, quantity and bacterial degradation of OM produced by a scleractinian coral, and to link these changes to coral metabolism. *A. muricata* was chosen because it belongs to one of the 10 most abundant genera in New Caledonia (Fenner and Muir, 2008) and worldwide (Veron, 2000), and is likely to be one of the major contributors affecting the reef biogeochemical processes. We hypothesize that UVR will exacerbate the effect of thermal stress on coral bleaching and overall metabolism. We also hypothesize that each stressor, alone or in combination, will alter organic carbon and nitrogen fluxes, both in terms of quantity and quality, which will likely change bacterial enzymatic activity in the released mucus. These changes will have a cascading effect on the whole pattern of reef nutrient recycling under global warming scenario.

## RESULTS

### Effects of temperature and UVR on coral physiology

**Fig. 1.**
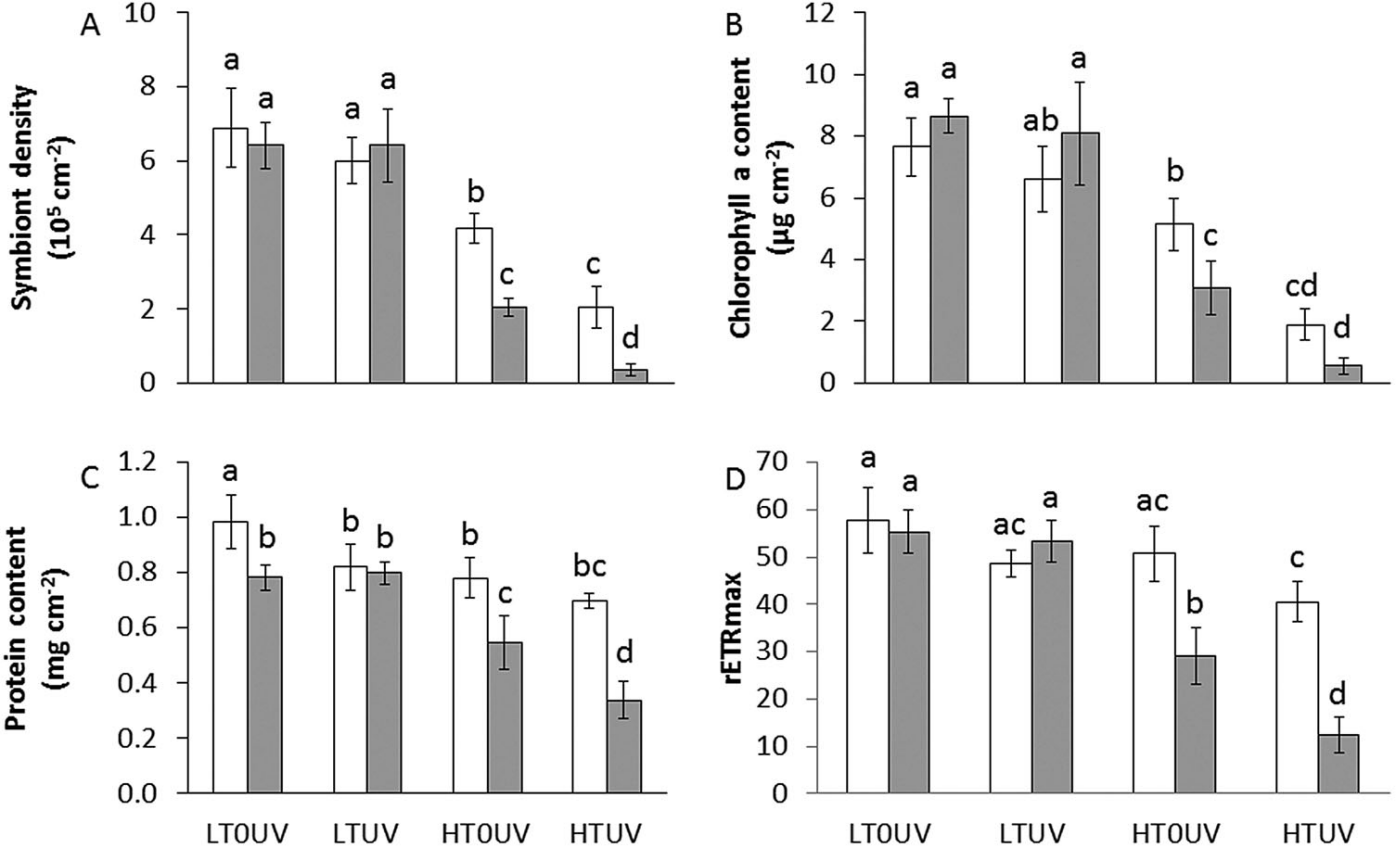
**Tissue parameters of *A. muricata* after 16 and 28 days of thermal stress with or without UVR.** Symbiont density (A), chl *a* content (B), protein content (C) and rETR_max_ (D) assessed at 26°C without UVR (LT0UV), 26°C with UVR (LTUV), 30°C without UVR (HT0UV) and 30°C with UVR (HTUV) after 16 (white columns) or 28 (grey columns) days of thermal stress. Data are mean±standard deviation (s.d.) of five replicates. Values with the same letter are not significantly different (*P*>0.05).

**Fig. 2.**
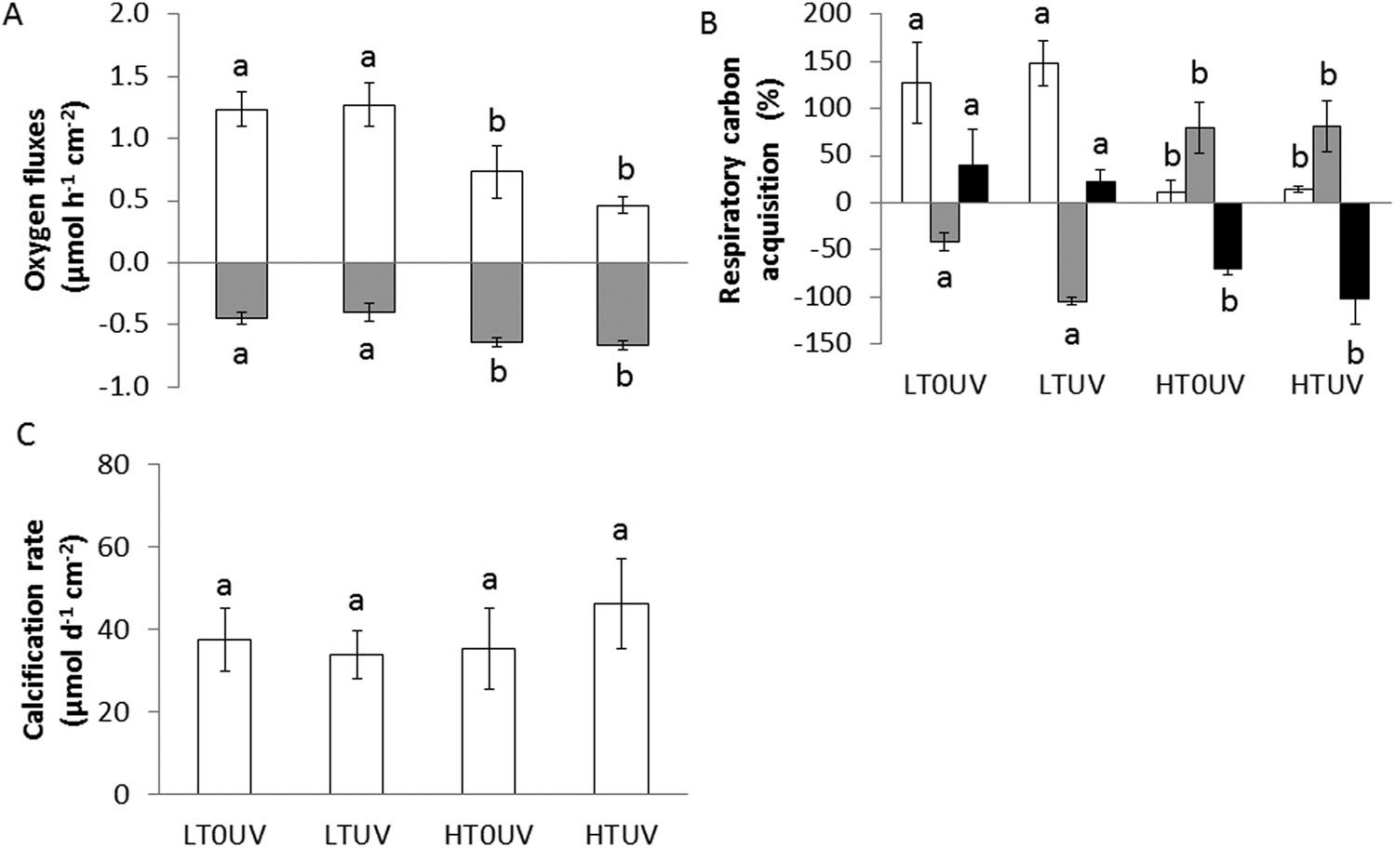
**Metabolism of *A. muricata* after 16 days of thermal stress with or without UVR.** Oxygen fluxes (A), respiratory carbon acquisition (B), and calcification rate (C) after 2 weeks of thermal stress measured at 26°C without UVR (LT0UV), 26°C with UVR (LTUV), 30°C without UVR (HT0UV) and 30°C with UVR (HTUV). In A, white columns represent the net photosynthesis, grey columns represent respiration rates. Data are mean±s.d. of five replicates. In C, white columns are autotrophic carbon (CZAR), grey columns are CHAR_POC_ and black columns are CHAR_DOC_. Values with the same letter are not significantly different (*P*>0.05).

Four conditions were tested: 26°C without UVR (LT0UV), 26°C with UVR (LTUV), 30°C without UVR (HT0UV) and 30°C with UVR (HTUV) (see Materials and Methods). After 2 weeks at 26°C, exposure to UVR (LTUV) had no significant effect on coral physiology, except for the protein concentration, which was significantly lower in UV treatment (Figs 1 and 2; Table S1). Conversely, exposure to elevated temperature alone (HT0UV) impaired the symbiont density, the chlorophyll *a* (chl *a*) and protein content, and the maximum relative electron transport rate (rETR_max_) (Fig. 1; Table S1). The combination of both stressors had an interactive (protein and rETR_max_) or synergistic (symbiont density and chl *a*) impact on coral physiology (HTUV; Table S1). Two weeks of thermal stress alone induced a 30% and 40% decrease in symbiont density and chl *a*, respectively, and this decline reached 70% and 60%, respectively, when thermal stress was combined with UVR exposure (Fig. 1A,B; Table S1). Net photosynthesis (Pn) normalized to the nubbins' surface area following the same trend as chl *a* (40% and 60% decrease in HT0UV and HTUV), while respiration rates increased by 40% in both treatments compared to 26°C (Fig. 1B and Fig. 2A; Tables S1 and S2). As photosynthesis slowed down and temperature increased under high temperature, the contribution of symbionts (Zooxanthellae) to the animal respiration (CZAR) significantly decreased from 137±33% in control, ambient temperature conditions (LT0UV or LTUV), to *ca.* 13±7% under both high temperature conditions (HT0UV or HTUV) (Fig. 2B; Table S2). However, after 2 weeks of thermal stress, no significant difference was observed between treatments in the rETR_max_ (Fig. 1D, Tuckey's test), which was reached at 600 µmol photons m^−2^ s^−1^. Photosynthetic apparatus was therefore not affected by the combination of stressors after 2 weeks. Despite significant changes in photosynthesis, calcification rates were not significantly different between conditions (Fig. 2C; Table S2).

After 4 weeks of thermal stress, symbiont density, chl *a* and protein content significantly decreased compared to measurements performed after 2 weeks (synergistic effects of time and temperature, Table S1). The rETR_max_ also decreased showing that the photosynthetic apparatus is damaged by long-term exposure to stress. A synergistic effect between high temperature and UVR was also notable after 4 weeks of stress (Table S1). Therefore, corals lost 70% of their symbionts and chl *a* content in the HT0UV treatment, and almost 95% in the presence of UVR (HTUV) (Fig. 1A,B; Table S1). Their protein content declined by 30% and 50% in the HT0UV and HTUV treatments (Fig. 1C; Table S1), while the rETR_max_ dropped by 50% and 77%, respectively (Fig. 1D; Table S1). Despite a significant bleaching per surface area, the chl *a* content and Pn normalized per symbiont cell stayed constant under stress and over time (12.3±1.5 pg cell^−1^ and 17.7±7.6×10^−7^ µmol h^−1^ cell^−1^, respectively) (Tables S1 and S2).

### Heterotrophy and organic matter release

**Fig. 3.**
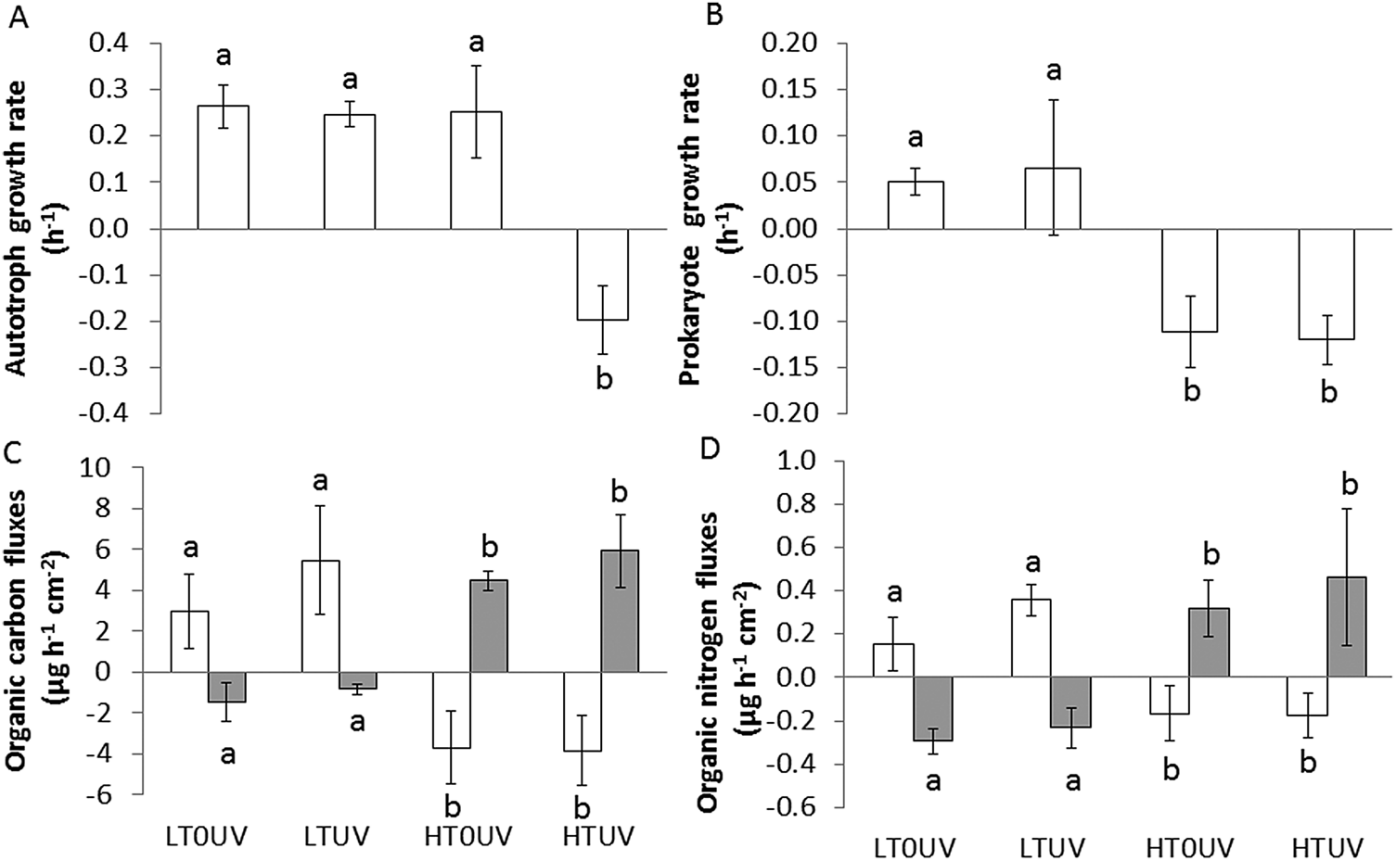
**Growth rates of the mucus-associated micro-organisms and organic matter fluxes.** Autotroph growth rate (A), prokaryote growth rate (B), organic carbon fluxes (C) and organic nitrogen fluxes (D) at 26°C without UVR (LT0UV), 26°C with UVR (LTUV), 30°C without UVR (HT0UV) and 30°C with UVR (HTUV) after 2 weeks of thermal stress. In C and D, white columns are POM and grey columns are DOM. Data are mean±s.d. of five replicates. Values with the same letter are not significantly different (*P*>0.05).

At 26°C (LTUV and LT0UV), autotrophs and prokaryotes presented positive and similar growth rates during the 4 h incubation. However, negative growth rates were observed for prokaryotes in HTUV and HT0UV conditions, and for autotrophs in the HTUV treatment (synergistic effect of UVR and temperature) (Fig. 3A,B; Table S2). These negative rates were linked to their ingestion by coral nubbins at a mean rate of 1.6±1×10^5^ prokaryotes h^−1^ cm^−2^ and 4.6±1.7×10^3^ autotrophs h^−1^ cm^−2^, respectively.

Total organic matter (OM) fluxes were positive and not significantly different between treatments, showing a similar increase in total organic carbon (3.11±1.39 µg C cm^−2^ h^−1^) and nitrogen (0.24±0.09 µg N cm^−2^ h^−1^) concentrations in seawater. Particulate organic matter (POM) and dissolved organic matter (DOM) fluxes, however, showed inverse trends (Fig. 3C,D; Table S2), with positive fluxes for POM and negative fluxes for DOM at 26°C and the opposite trend at 30°C. There was, therefore, a significant temperature effect on the POM and DOM fluxes (Table S2), while no significant effect of UVR exposure was detected. Dissolved organic carbon (DOC) uptake at 26°C contributed to 39% of the daily respiratory needs (CHAR_DOC_), while particulate organic carbon (POC) uptake at 30°C contributed to 80% of the respiratory needs (CHAR_POC_).

### Enzymatic activity and organic matter degradation

**Fig. 4.**
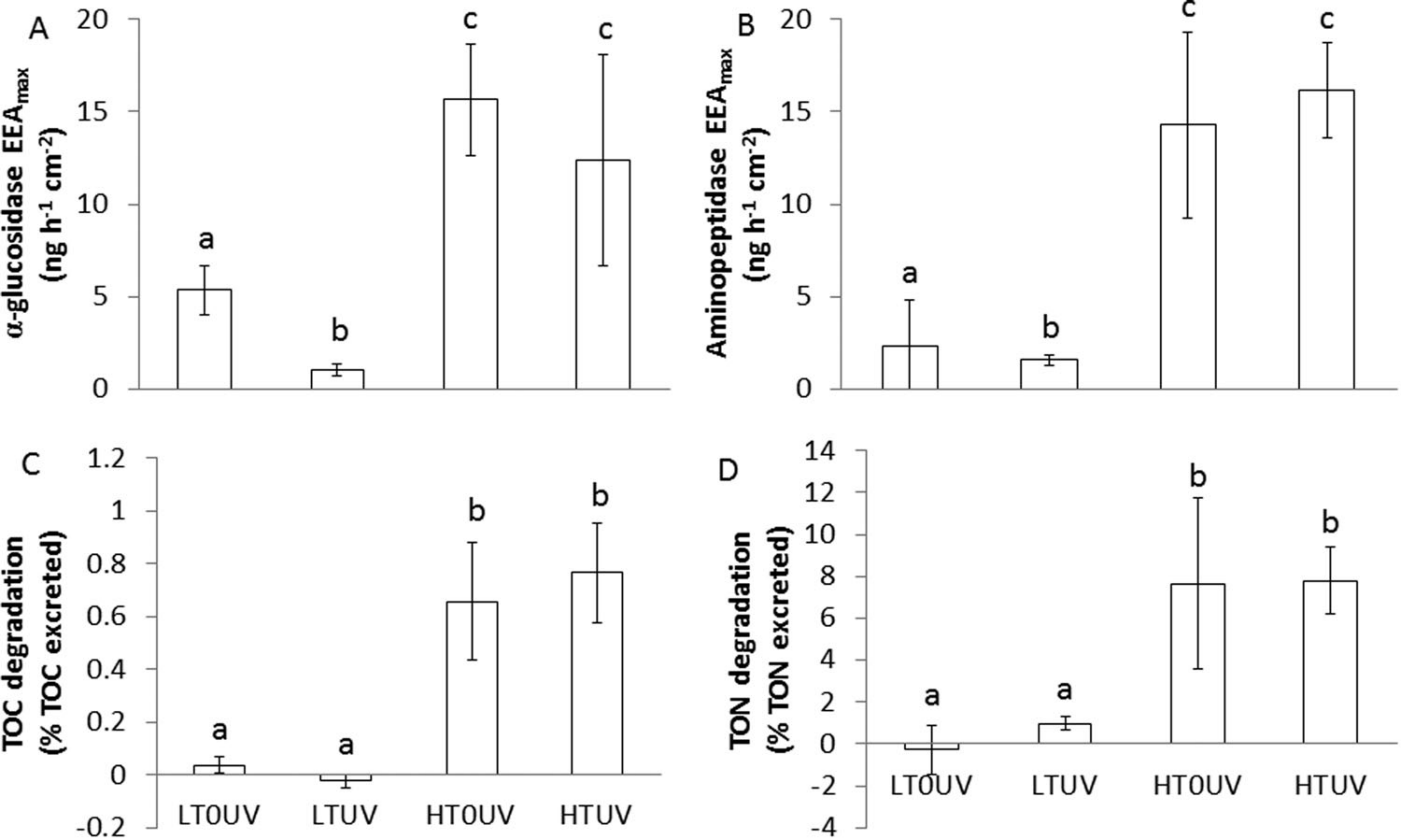
**Bacterial activity and organic matter degradation.** Alpha-glucosidase EEA_max_ (A), aminopeptidase EEA_max_ (B), TOC degradation (C) and TON degradation (D), calculated at 26°C without UVR (LT0UV), 26°C with UVR (LTUV), 30°C without UVR (HT0UV) and 30°C with UVR (HTUV) after 2 weeks of thermal stress. Data are mean±s.d. of five replicates. Values with the same letter are not significantly different (*P*>0.05).

At 26°C, glucosidase maximum extracellular enzyme activity (EEA_max_) was significantly higher in nubbins shaded from UVR (5.4±1.3 ng h^−1^ cm^−2^, Fig. 4A). High temperatures significantly increased glucosidase EEA_max_, which reached 15.2±3.0 ng h^−1^ cm^−2^ (Fig. 4A; Table S2), without any effect of UVR (Table S2). Aminopeptidase EEA_max_ presented the same trends as the glucosidase EEA_max_, with low rates at 26°C (Fig. 4B, 2.3±2.3 ng h^−1^ cm^−2^) and a significant increase at 30°C (16.1±2.5 ng h^−1^ cm^−2^) (Table S2). UVR exposure had no significant effect on this activity.

At low temperature, only a small fraction of the total OM released was degraded (0.03% and 1% of the total carbon and nitrogen released, respectively). However, at high temperature, 0.7% and 8% for the total carbon and nitrogen released were degraded, respectively (Fig. 4C,D; Table S2).

## DISCUSSION

By simultaneously quantifying covariation in coral photosynthesis, calcification, tissue composition, OM fluxes and degradation by mucus-associated bacteria, this study allows deeper understanding of how two major environmental factors, elevated temperature and UVR, alone or in combination, impact the metabolism and close surrounding environment of *A. muricata*, a dominant coral reef species (Fenner and Muir, 2008). Specifically, our results demonstrate that elevated temperature was the main factor to affect the overall metabolism of *A. muricata*, as well as OM fluxes and bacterial activity. The results, however, highlight a significant combined effect of UVR and temperature on the bleaching susceptibility and photosynthetic efficiency of this coral species, as well as on the decrease in protein reserves over time. This study also reveals, for the first time, that the shift from auto- to heterotrophy that occurred in the short-term bleached *Acropora* nubbins led to a change in both the quality of the OM released and the population dynamics of the associated microorganisms. Finally, OM degradation by mucus-associated bacterial activity was unaffected by UVR exposure, but significantly increased under high temperature. Altogether, our results demonstrate that seawater warming not only affects coral physiology, but also the way corals interact with their nearest environment, with potential consequences for coral reef biogeochemical cycles and food webs.

*A. muricata* nubbins did not present any change in their physiology when they were experimentally shaded from UVR and maintained under their normal growth temperature. This lack of UVR effect was likely due to the acclimation to UVR of the colonies used in this experiment, which grew at 2-3 m depth and were therefore likely protected by mycosporine-like amino acids (MAAs), synthesized in most shallow water corals (Shick et al., 1995). This protection was, however, suppressed or reduced under thermal stress (Fitt and Warner, 1995), lowering the coral's capacity to cope with the accumulation of reactive oxygen species and oxidative stress (Lesser et al., 1990). Temperature presented an additive and synergistic effect with UVR on symbiont density and chl *a* content, respectively. Corals, therefore, bleached and lost 35% of their symbionts under thermal stress alone, and up to 68% under the combined stress, consistent with previous laboratory and field studies which showed greater effects of double than single stress in diverse coral species (Courtial et al., 2017; D'Croz and Maté, 2002; Ferrier-Pagès et al., 2007; Fitt and Warner, 1995). Bleaching was amplified with the stress duration as only 5% of the symbionts remained in nubbins kept for 4 weeks at high temperature under UVR. These results suggest that corals naturally exposed to low UVR could better resist long thermal stress events than UVR-exposed corals.

In addition to studying the effects of thermal and UVR stress on coral physiology, we also expanded our measurements to assess the coral-induced changes in seawater biogeochemistry (via mucus release and degradation) with thermal and/or UVR stress. Coral mucus (i.e. dissolved and particulate organic carbon and nitrogen) has several functions, both for corals (defense against external stressors and food source (Brown and Bythell, 2005; Levas et al., 2016) and for the reef organisms (energy carrier and particle trap) (Wild et al., 2004); however, changes in mucus quality and quantity under different environmental conditions are still poorly understood (Niggl et al., 2008; Tremblay et al., 2012), because few studies have investigated both carbon and nitrogen fluxes, in dissolved or particulate forms, in healthy and stressed coral species (Bednarz et al., 2012; Naumann et al., 2010), preventing a comprehensive overview of OM fluxes in corals. Our results first show constant release rates of total organic carbon (TOC) and total organic nitrogen (TON) by *A. muricata*, irrespective of the stress state. The high TOC/TON ratio (13 to 15) of the released OM, already observed for several coral species of the Red Sea (Naumann et al., 2010), indicates a higher degree of nitrogen retention in coral tissue compared to carbon. Although *A. muricata* also presents 10 times higher TOC release rates than other species of the Red Sea or the Caribbean (Levas et al., 2016; Naumann et al., 2010), these rates are in the range of previously reported values in several *Acropora* species from Malaysia and Jordan (Nakajima et al., 2009, 2010; Naumann et al., 2010). Overall, *Acropora* species tend to mainly be a source of energy-rich carbon compounds to the reef food chain. In New Caledonia, this production can partly explain the exceptionally high rates of N_2_ fixation in the water column (Camps et al., 2016) compared to other reef systems (Bednarz et al., 2017), since diazotrophs need large amounts of energy-rich photosynthates to perform N_2_ fixation (Bednarz et al., 2017).

The analysis of the DOM and POM forms shows that the quality of the OM (i.e. particulate or dissolved matter) changes with the environmental conditions under which corals are thriving. *A muricata* released POM and took up DOM under normal growth conditions, while the reverse was observed in bleached colonies. Although most previous studies show a release of DOC by healthy corals (Crossland, 1987; Wild et al., 2010a,b, 2004a,b, 2005, 2008; Houlbrèque et al., 2004; Tanaka et al., 2008, 2009; Haas et al., 2010; Naumann et al., 2010; Levas et al., 2015), some studies show the contrary (Houlbrèque et al., 2004; Naumann et al., 2010; Niggl et al., 2008). Observations of OM fluxes in bleached or thermally-stressed corals evidenced the same contrasted results: while *Porites divaricata*, *Porites astreoides* and *Orbicella faveolata*, were shown to take up DOC (Grottoli et al., 2006, 2014; Levas et al., 2013, 2016), *Acropora* sp., *Porites* spp. and *Stylophora pistillata* released it (Haas et al., 2010; Niggl et al., 2008; Tremblay et al., 2012). Overall, no common pattern can be deduced from these previous observations. Our measurements, which took into account the OM as well as the pico- and nanoplankton concentrations, however, suggest that the changes in POM and DOM fluxes in *A. muricata* are linked to its heterotrophic activity. POM includes both nonliving material and living particles such as bacteria and small autotrophs contained or grown in the mucus. Pico- and nanoplankton, which multiplied in the incubations with healthy coral colonies, were instead grazed by corals when bleached. A shift thus occurred between low-energy DOM uptake under healthy conditions (maximum of 1.5 µg C and N h^−1^ cm^−2^ when all DOM is consumed) to high energy POM uptake under bleached conditions, (maximum of 3.9 µg C and N h^−1^ cm^−2^ when all POM is consumed), suggesting a greater need for heterotrophic nutrients, likely to compensate for autotrophic loss and meet metabolic demand. POM uptake contributed 80% of the respiratory needs of the heat-stress colonies, compared to 40% for DOM under healthy conditions. In addition, POM consumption in bleached corals may have enhanced DOC release via sloppy feeding. Although this process was never studied in corals, it is well-known in copepods as a dominant mode of DOM production (Saba et al., 2011). Moreover, our results highlight a positive correlation between the stress level inflicted to the corals and their micro-heterotrophy level: corals shifted from total autotrophy under healthy conditions to partial heterotrophy on prokaryotes alone under thermal stress, and to predation on both prokaryotes and autotrophs when exposed to thermal and UVR stress. These results demonstrate the role of pico/nanoplankton food sources for *A. muricata* resilience to thermal stress, and for bleached corals in general (Houlbrèque and Ferrier-Pagès, 2009; Tremblay et al., 2012). They also clearly indicate that corals can feed on allochtonous aggregates (Coffroth, 1984), and on their own mucus and mucus associated particles, when needed. The ability to shift from autotrophy to heterotrophy is believed to provide significant advantage over species that are unable to do so (Hughes and Grottoli, 2013; Levas et al., 2016). In this study, high temperature induced a decrease in the rates of photosynthesis, without any impact on calcification, although the two processes are usually correlated (Gattuso et al. 1999). The shift to heterotrophy at high temperature indeed allowed *A. muricata* to increase its respiration rates, likely to keep up with energy costs associated with the reparation of damages caused by thermal stress (Coles and Jokiel, 1977; Fitt et al., 2001). This increased production of internal CO_2_ likely sustained calcification rates, since more than 70% of the CO_2_ used in calcification come from internal respiration (Furla et al., 2000).

Since bacteria are the first consumers of the carbon-rich compounds (i.e. wax esters, triglycerides, fatty acids) contained in the mucus, to convert them into bacterial biomass (Ferrier-Pagès et al., 1998; Herndl and Velimirov, 1986), we quantified in the different temperature and UV conditions, the activity of the two main bacterial enzymes responsible for carbon (α-glucosidase) and nitrogen (aminopeptidase) degradation. Shading nubbins from UVR had little effect on enzymatic activities, which contrasts with previous studies performed *in vitro* with isolated enzymes, showing a decrease in EEA under UVR because of photolysis (Espeland and Wetzel, 2001). MAAs release by corals in the mucus (Drollet et al., 1997) might have protected the associated bacteria and enzymes from UVR damage. On the other hand, and consistent with observations made on water column bacteria (Cunha et al., 2010; Price and Sowers, 2004), high temperature enhanced both aminopeptidase and α-glucosidase EEA, either directly or indirectly through increased bacterial concentration. As a consequence, carbon and nitrogen degradation rates were 20 and 10 times higher, respectively, than at normal temperature. Despite this large increase in OM degradation, the matter degraded by mucus-associated bacteria represented <1% of the carbon and 10% of the nitrogen contained in the excreted mucus. This shows that the recycling of the coral derived-matter is a long-term process, rather performed by bacteria free living in the water column or in reef sediment. Nitrogen was overall 10 times more degraded than carbon, likely because it is one of the major nutrients limiting bacterioplankton growth (Antia et al., 1991; Keil and Kirchman, 1991).

**Fig. 5.**
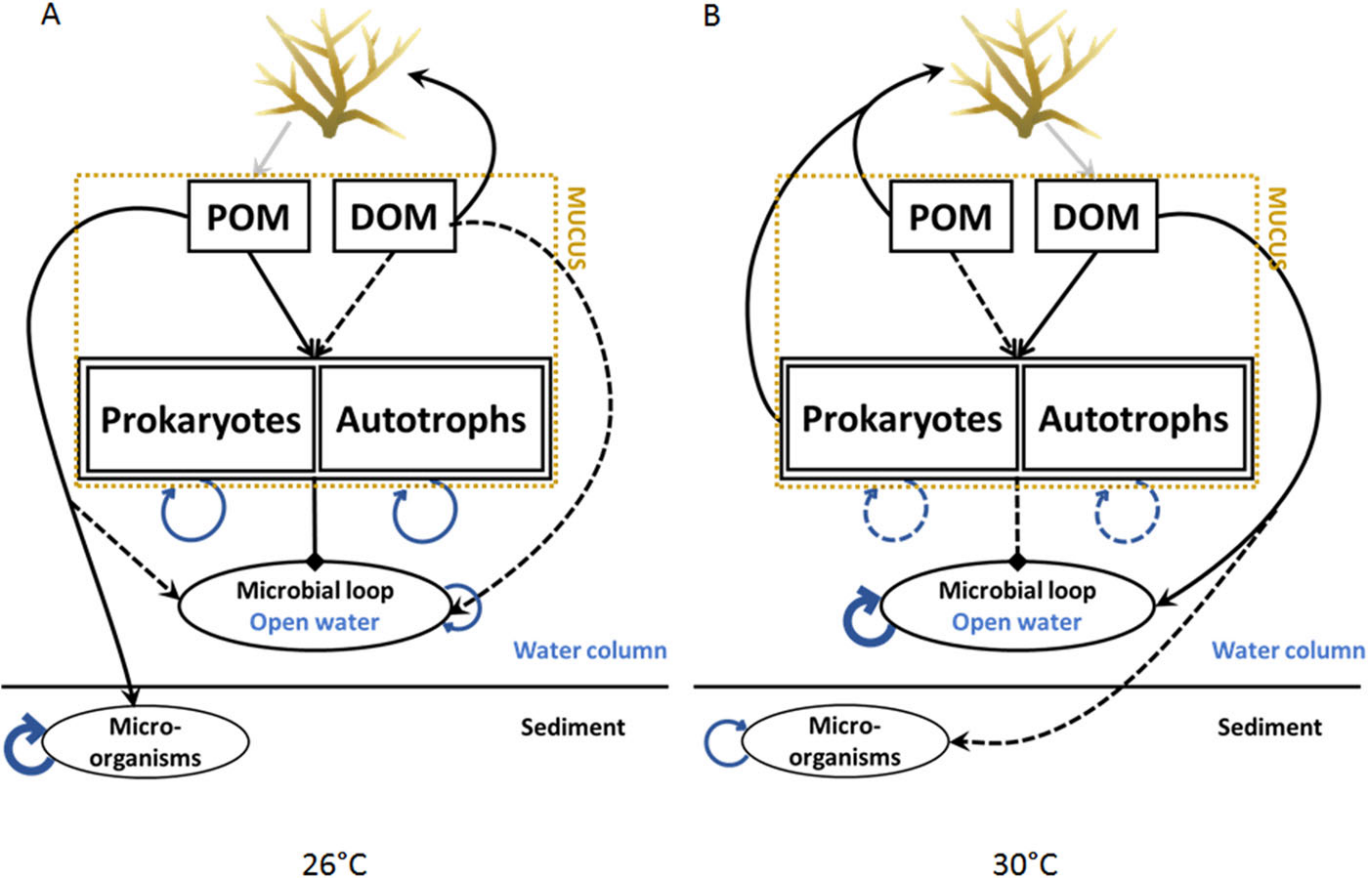
**Summary of organic matter exchange between coral microbial communities.** Uptake (black arrows) and release (grey arrows) fluxes between coral nubbins-associated micro-organisms and near seawater at 26°C (A) and 30°C (B). Blue arrows indicate organisms' growth. Diamond-headed lines (from ‘Prokaryotes/Autotrophs’ to ‘Microbial loop/Open water’) indicate the contribution of mucus-associated microbiome to the open water microbial loop. Dashed lines indicate low fluxes, solid lines indicate enhanced processes. At normal temperature (26°C), coral releases POM and ingests DOM. Prokaryotes and autotrophs grow in the mucus and contribute to the open water microbial loop. The released POM sinks and is then available for the development of the microbial loop inside sediments and in open water. At high temperature (30°C), corals ingest POM and release DOM. They graze on associated microorganisms, which contribute less to the microbial loop of the water column. Overall, DOM is released to the seawater which enhances the development of microorganisms in the water column.

Overall, our study highlights the major changes in OM fluxes, composition and degradation following *A. muricata* bleaching. The potential organic carbon and nitrogen pathways expected with healthy (A) and bleached (B) *A. muricata* are represented in Fig. 5. Under healthy conditions, *A. muricata* releases POM, which is poorly degraded by prokaryotes. POM will thus rapidly sediment to the reef bottoms, where it will sustain bacterial growth, and will contribute to the important nutrient recycling pathways observed in reefs (Muscatine and Porter, 1977; Richter et al., 2001; Wild et al., 2004). During bleaching, *A. muricata* will release labile DOM, which is more likely to stay in the water column and therefore promote the development of free-living-bacteria (Ferrier-Pagès et al., 2000; Wild et al., 2004), including pathogenic communities (Haas et al., 2013; Nelson et al., 2013). Unless bacteria enter the microbial loop and higher trophic levels, such stimulation may lead to the ‘microbialization’ of the reef (Haas et al., 2016) with negative consequences for coral health such as the promotion of opportunistic pathogen invasion (Barott and Rohwer, 2012). Our study also emphasizes the importance of considering UVR exposure when predicting long-term coral bleaching. As UVR impact on coral physiology is increased with the stress duration, the effects measured during this short-term experiment could be underestimated on a longer term.

## MATERIALS AND METHODS

### Coral collection and experimental setup

Eighty 2-3 cm long *A. muricata* nubbins were collected between 2 and 3 m depth from 10 parent colonies on the reef of ‘phare Amédée’ in the New Caledonian lagoon (22°28.845′S; 166°26.806′E) in April 2016 (APA-NCPS-2016-001). Nubbins were then transferred at the Aquarium des Lagons in Nouméa and left to recover for 1 month prior to the experiment. They were evenly distributed in eight 100 L outdoor tanks continuously supplied with the lagoon seawater at a rate of 72 L h^−1^, and were fed once a week with *Artemia salina* nauplii. Shade cloths were disposed above aquaria to reach the underwater light conditions on the reef. Therefore, at the beginning of the experiment, the maximal natural irradiances in aquaria, obtained at midday, were ∼900 µmol photons m^−2^ s^−1^ of photosynthetically active radiation (PAR), and ∼20 W m^−2^ and ∼1.2 W m^−2^ of ultra-violet A (UVA, 315-400 nm) and B (UVB, 280-315 nm) radiation, respectively. PAR was controlled using a LI-COR data logger (LI-1000, LI-COR, Lincoln, USA) connected to a spherical quantum sensor (LI-193, LI-COR); UVR was controlled using an ILT1400 portable radiometer (International Light Technologies, Peabody, USA) connected to detectors (SEL240/UVB-1/TD and SEL033/UVA/TD, International Light Technologies). In addition, during the 1 month acclimation period, four tanks were maintained under the above natural conditions (UV), while the other four tanks were shielded from UVA and UVB using UVR filters (226 Lee UV filters, La Boutique du Spectacle, Paris, France) (0UV).

After the acclimation period, for each treatment (0UV and UV), two temperature levels were set with duplicated tanks: 26°C (±0.5°C), corresponding to a natural condition, and 30°C (±0.5°C), corresponding to a heat-stress condition. Four conditions were thus reached in a cross factor design: 26°C without UVR (LT0UV); 26°C with UVR (LTUV); 30°C without UVR (HT0UV); 30°C with UVR (HTUV). For high-temperature (HT) conditions, temperature was gradually increased from 26°C to 30°C within 1 week, and was controlled using heaters connected to a temperature-stat system (IKS, Karlsbad, Germany; accuracy ±0.05°C). Two samplings were performed after 16 days and 28 days incubation under the different conditions (for both 26°C and 30°C, with or without UVR). After 16 days, five nubbins per condition and for all conditions (LT0UV, LTUV, HT0UV, HTUV), randomly taken in the replicated tanks, were used to assess their photosynthetic performances (i.e. rETR_max_, photosynthetic and respiration rates) and growth rates and then frozen at −20°C for symbiont, chl *a*, protein content and surface measurements. In the meantime, five additional nubbins per condition were used for OM fluxes and bacterial activity measurements (as described below) before being frozen at −20°C. Twelve days later (28 days of stress), the physiological parameters (chl *a*, symbiont density, protein and rETRmax) were re-assessed on the remaining nubbins (five per condition and for all conditions).

For all incubations, temperature and light were maintained constant and identical to the experimental conditions. All data were normalized to the surface area of the corals, measured using the wax technique (Stimson and Kinzie, 1991), or per symbiont cell (determined as described below).

### Physiological measurements

Five nubbins per condition were incubated in the dark for 10 min before the relative electron transport rate (rETR) versus irradiance, or rapid light curves, were generated with a Pulse Amplitude Modulation (PAM) fluorometer (Diving-PAM, Walz, Effeltrich, Germany) according to Ralph and Gademann (2005). For this purpose, nubbins were illuminated for 10 s with seven different light intensities (from 0 to 900 µmol quanta m^−2^ s^−1^) and the rETR_max_ were deduced from the curves.

The same nubbins were then used to measure rates of photosynthesis, respiration and calcification. They were thus incubated in individual 100 ml beakers filled with 0.45 µm filtered seawater, continuously stirred with stirring bars and hermetically closed using transparent plastic film to avoid any oxygen exchange with the ambient air. Each beaker was equipped with an oxygen sensor spot (SP-PSt6-NAU, PreSens, Regensburg, Germany), and oxygen concentration was measured with a polymer optical fiber and Fibox 4 (PreSens) at the beginning and at the end of the incubations. Changes in oxygen production were measured during 1 h incubation in the dark for the determination of the respiration rate (R) and 30 min at the optimal photosynthetic light (600 µmol photons m^−2^ s^−1^, Aquablue plus neon, Blue-white, 15,000 K, Giesemann, Nettetal, Germany) to assess net photosynthesis (Pn). This optimal irradiance was deduced from the rapid light curves and from preliminary Pn-irradiance curves (Fig. S1). Pn and R were estimated from the difference between the final and the initial oxygen concentrations. After the incubations, nubbins were frozen at –20°C for the subsequent determination of tissue parameters as described below. Data were expressed in µmol O_2_ h^−1^ cm^−2^ d^−1^ and corrected against a blank (filtrated seawater incubated for the same period without nubbin).

The incubation seawater of each beaker was filtered through 0.2 µm filters and stored at 4°C for the subsequent determination of the calcification rates using the total alkalinity anomaly method (Smith and Key, 1975). The measurement was performed using a TIM865 titration manager (TitraLab, Hach, Loveland, USA). Titration error was verified using AT standards provided by A.G. Dickson (University of California, San Diego, USA; batch 142). Data were expressed in µmol CaCO_3_ cm^−2^ d^−1^ and corrected against a blank (filtrated seawater incubated for the same period without nubbin).

To calculate the total daily CZAR, oxygen fluxes were converted to carbon equivalent using respiratory (RQ) and photosynthetic (PQ) quotients equal to 0.8 and 1.1, respectively (Gattuso and Jaubert, 1990; Muscatine et al., 1981). We considered that coral colonies photosynthesize during 12 h (light period) and respire during 24 h. CZAR (%) was calculated as(1)
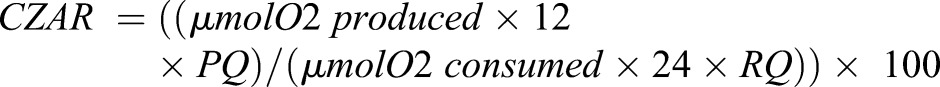
Here, we considered the respiration of the whole coral holobiont (i.e. host, symbionts and microbiome), not only the animal, to take into account all respiratory needs.

### Symbiont, chl *a* and protein content

Nubbin tissue was removed from the skeleton using an air pick and homogenized with a Potter tissue grinder. A sub-sample was taken for the determination of the symbiont density of each sample using a Neubauer cell, on five replicated counts. Another sub-sample was used to assess the protein content according to Hoogenboom et al. (2010), using a BCA assay kit (Smith et al., 1985). For chl *a* measurements, the last subsample was centrifuged at 5000×***g*** for 10 min at 4°C to separate the symbionts (in the pellet) from the host tissue. The pellet was then re-suspended in 10 ml acetone and kept in the dark at 4°C for 24 h prior measurements. Samples were then centrifuged for 15 min at 10,000×***g*** and the absorbance was measured at 630, 663 and 750 nm using an EVOLUTION 201 UV-Visible spectrophotometer (Thermo Fisher Scientific). Chlorophyll concentrations were computed using the equations of Jeffrey and Humphrey (1975).

### POM and DOM fluxes

Five nubbins per condition were incubated for 4 h in 200 ml of 0.45 µm filtered seawater. Water samples were taken at the beginning (T_0_) and at the end (T_f_) of the incubation to assess (1) the organic carbon and nitrogen fluxes, (2) the abundance of heterotrophic prokaryotes and autotrophs in the organic matter released by the corals and (3) the EEA. All measurements were corrected against a blank (filtered seawater incubated without nubbin).

#### Organic carbon and nitrogen fluxes

For the determination of total organic carbon and nitrogen concentrations in the incubation medium at T_0_ and T_f_, 20 ml seawater were sampled at T_0_ and T_f_ in each beaker with sterile syringes and transferred to glass vials, previously washed for 24 h in 10% HCl and burned at 500°C for 4 h (Naumann et al., 2010). Vials were amended with 42 µl H_3_PO_4_ to avoid any bacterial activity during storage, and they were then stored at −20°C until further analysis. The same procedure was applied to measure DOC and DON concentrations, except that the water was filtered through 0.45 µm filters prior to storage. Samples were analyzed using a TOC-L analyzer (Shimadzu, Kyoto, Japan).

To calculate the TOC/TON and DOC/DON fluxes, concentrations of each compound were first corrected from the blank (concentrations in beakers incubated without a coral nubbin), and the difference between T0 and Tf was then calculated to deduce a flux between corals and seawater. Negative fluxes indicate a net uptake by coral nubbins, and positive fluxes indicate a net release from corals. In addition, POC and PON were estimated by subtracting DOC or DON from TOC or TON. Data were expressed in µg h^−1^ cm^−2^.

To calculate the total daily heterotrophic contribution of POC and DOC to the animal respiration (CHAR_POC_ and CHAR_DOC_, respectively), carbon fluxes were converted to µmol C h^−1^ cm^−2^ and 0.8 was used as a respiratory quotient (RQ) to convert oxygen fluxes to their carbon equivalent (Gattuso and Jaubert, 1990; Muscatine et al., 1981). CHAR (%) was calculated as(2)
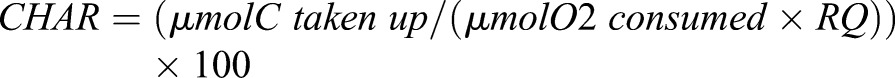


#### Abundance of pico-and nanoplankton

For the determination of the abundance of heterotrophic prokaryotes and autotrophs, 4.8 ml seawater was sampled at T_0_ and T_f_, fixed with 0.2 ml glutaraldehyde (25%) during 30 min in the dark, and stored at −80°C. Samples were then analyzed by flow cytometry as described by Jacquet et al. (2013). Particle growth rates or grazing rates were calculated according to the equations of Ribes et al. (1998). In brief, growth rates in the control beakers (k_c_) and in the presence of coral nubbins (k_n_) were computed as:(3)
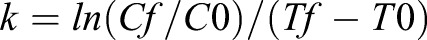
with C_0_ and C_f_ representing the microorganism concentrations (cell ml^−1^) at time T_0_ and T_f_ , respectively, expressed per hour. These growth rates were then used to calculate g, the grazing coefficient (h^−1^),(4)
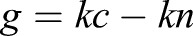
They were also used to calculate F, the filtration rate (ml h^−1^ cm^−2^)(5)
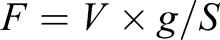
where V is the incubation volume (ml), S the coral surface (cm^2^) and g (h^−1^) the grazing coefficient. The ingestion rate I (ingested cell h^−1^ cm^−2^) was finally calculated as(6)
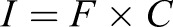
where F (ml h^−1^ cm^−2^) is the filtration rate and C (cell ml^−1^) is the average prey concentration equal to(7)
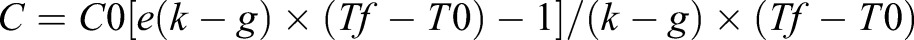
with C_0_ the prey concentration at T_0_. Because of the heterogeneity in the carbon content of the different autotrophs present in seawater, we could not quantify the equivalent carbon ingested by the coral using these data. However, this contribution was estimated using the CHAR_POC_ above.

#### Extracellular enzymatic activities

EEAs were monitored using fluorescent substrate analogs according to standard protocols (Hoppe, 1983). L-Leucine-7-amido-4-methyl-coumarin hydrochloride (Leu-MCA) and 4-methylumbeliferyl-α-D-glucopyranoside (αMUF) were therefore used to assess aminopeptidase and alpha-glucosidase activities, respectively. These two compounds emit a fluorescent signal after cleavage by the enzymes. Correspondence between fluorescence value and the quantity of substrate hydrolyzed was obtained using amino 4-methylcoumarin (MCA) and 4-methylumbeliferone (MUF). Three replicates of the incubation water were sampled at the end of the 4 h incubation and added to the substrates to a final concentration of 250 µM. Samples were incubated in the dark for up to 168 h in a thermostated shaking bath at the experimental temperatures (26°C or 30°C). EEAs were measured every 24 h using a spectrofluorometer (Synergy-H1, BioTek, Bad Friedrichshall, Germany). For this purpose, 96-well plates (300 µl volume) were filled up with 240 µl sample and 60 µl of 10.8 Tris-HCl buffer was added to adjust pH to obtain maximal fluorescence intensity (Fonvielle et al., 2015).

Carbon degradation was calculated with the following equation:(8)
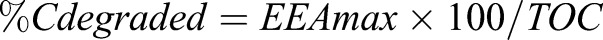


with EEA_max_ the α-glucosidase maximal enzymatic activity (nmol l^−1^ h^−1^) of the mucus-associated bacteria and TOC (nmol l^−1^ h^−1^) the quantity of organic carbon contained in the mucus, calculated as(9)
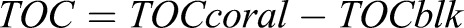


TOC_coral_ and TOC_blk_ (nmol l^−1^ h^−1^) are the TOC concentrations in seawater after 4 h of incubation with or without nubbin, respectively.

Similarly, nitrogen degradation was calculated using the following equation:(10)
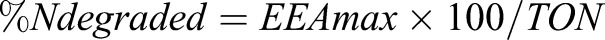


with EEA_max_ the aminopeptidase maximal enzymatic activity (nmol l^−1^ h^−1^) of the mucus-associated bacteria and TON (nmol l^−1^ h^−1^) the quantity of total organic nitrogen contained in the mucus, calculated as(11)
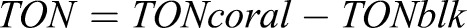


with TON_coral_ and TON_blk_ (nmol l^−1^ h^−1^) the TON concentrations in seawater after 4 h of incubation with or without nubbin, respectively.

### Statistical analysis

The effects of UVR and high temperature on the parameters measured after 16 days of thermal stress were assessed with two-way ANOVAs using UVR (presence, absence) and temperature (26°C and 30°C) as factors. To test the effects of high temperature and UVR over time, three-way ANOVAs using time (after 16 and 28 days), UVR and temperature as factors were performed on symbiont density, chl *a*, protein content and rETR_max_. For all tests, normality of the residuals and variance homoscedasticity were tested using Shapiro and Bartlett tests, respectively. When needed, data were log transformed in order to fulfill those criteria. A Tukey's post hoc test was performed when results of the ANOVAs were significant.
